# The Volunteer Medical Staff Corps at Walmer

**Published:** 1896-04-18

**Authors:** 


### THE VOLUNTEER MEDICAL STAFF CORPS AT WALMER.

(Communicated.)

The London companies of the Volunteer Medical Staff Corps, contrary to the usual practice at Eastertide of visiting the Royal Victoria Hospital at Netley, proceeded this year to Walmer, where they were quartered in the Royal Marine Barracks. The quarters were exceedingly comfortable. The programme of work differed slightly from the usual Easter course of training. Good Friday was devoted to route marching, Saturday to battalion and stretcher drills. Church parade was held early on Sunday morning, and was attended by the whole of the troops at the station. On Easter Monday, at the manœuvres in the neighbourhood of Shorncliffe, the corps provided two complete bearer companies, also a field hospital party, all of which were, in every detail, equipped to take the field, having a full complement of medical and surgical stores, tents, wagons, and water-carts. Although the zone of operations was necessarily more limited in extent than at Aldershot a considerable amount of valuable work was carried out. The bearer companies which followed up the fighting line during the day, as also the collecting and dressing-station parties, were under the command of Surgeon-Lieutenant J. Harper, the temporary field hospital (adapted from a farm-house, with its adjacent sheds and out-houses) being in charge of Surgeon-Captain J. Edward Squire. Here arrangements were made for the accommodation of a large number of sick—including a special ward for wounded officers—for the storing of patients' kit and effects, and the housing of the medical staff. Water supply, the provision of latrines, and other matters were attended to; dressers, compounders, and cooks were detailed. Communication was maintained throughout the lines of medical assistance by means of the regimental cyclists and signallers. The corps subsequently took part in the march past, and was complimented by Major-General Wood, C.B., on its remarkably steady marching and good appearance. The weather remained favourable, notwithstanding that the rain on Saturday night had rendered the chalky lanes somewhat unpleasant.

We may mention that Surgeon-Captain G. T. Rawnsley, A.M.S., is the new adjutant of the London Division, Surgeon-Captain D. M. O'Callaghan having vacated that appointment in December last on being seconded for service with the Ashantee expeditionary force.

